# Reconstructing Tuberculosis Services after Major Conflict: Experiences and Lessons Learned in East Timor

**DOI:** 10.1371/journal.pmed.0030383

**Published:** 2006-08-22

**Authors:** Nelson Martins, Paul M Kelly, Jocelyn A Grace, Anthony B Zwi

**Affiliations:** 1 Menzies School of Health Research, Darwin, Northern Territory, Australia; 2 Institute of Advanced Studies, Charles Darwin University, Darwin, Northern Territory, Australia; 3 National Centre for Epidemiology and Population Health, College of Medicine and Health Sciences, Australian National University, Canberra, Australian Capital Territory, Australia; 4 School of Public Health and Community Medicine, University of New South Wales, Sydney, New South Wales, Australia; The University of Queensland, Australia

## Abstract

**Background:**

Tuberculosis (TB) is a major public health problem in developing countries. Following the disruption to health services in East Timor due to violent political conflict in 1999, the National Tuberculosis Control Program was established, with a local non-government organisation as the lead agency. Within a few months, the TB program was operational in all districts.

**Methods and Findings:**

Using the East Timor TB program as a case study, we have examined the enabling factors for the implementation of this type of communicable disease control program in a post-conflict setting. Stakeholder analysis was undertaken, and semi-structured interviews were conducted in 2003 with 24 key local and international stakeholders. Coordination, cooperation, and collaboration were identified as major contributors to the success of the TB program. The existing local structure and experience of the local non-government organisation, the commitment among local personnel and international advisors to establishing an effective program, and the willingness of international advisers and local counterparts to be flexible in their approach were also important factors. This success was achieved despite major impediments, including mass population displacement, lack of infrastructure, and the competing interests of organisations working in the health sector.

**Conclusions:**

Five years after the conflict, the TB program continues to operate in all districts with high notification rates, although the lack of a feeling of ownership by government health workers remains a challenge. Lessons learned in East Timor may be applicable to other post-conflict settings where TB is highly prevalent, and may have relevance to other disease control programs.

## Abstract 2

A qualitative study of re-introduction of tuberculosis services in East Timor in 1999, after a period of civil conflict, concluded coordination, cooperation, and collaboration contributed to the success achieved.

## Editors' Summary

**Background.**

Tuberculosis is an infectious disease and one of the world's most serious health problems. It causes between 2 million and 3 million deaths every year, most of them in developing countries. The success of national control programs has varied considerably between countries. In times of war or other emergencies, control efforts are considerably hampered. East Timor is a former Portuguese colony in Southeast Asia annexed by Indonesia in 1975. It is a small country of about 1 million people situated some 500 miles northwest of Australia. In 1999, following a referendum on independence from Indonesia, violent civil conflict led to the destruction of much of East Timor's health-care system. As tuberculosis was known to be one of the country's biggest health problems, efforts to improve treatment were launched during the transition to independence in 2002. Several organizations, led by a local non-government organization (NGO), Caritas East Timor, collaborated in the new program. Many difficulties had to be overcome, including the forced movement of people away from their homes during the fighting, the departure of many health-care workers from the country, and the destruction of health-care facilities. Nevertheless, in its first three years the program diagnosed and commenced treatment on 10,722 patients. The rate of treatment success reached 81% in 2003, which—in international terms—is regarded as very high.

**Why Was This Study Done?**

The researchers wanted to find out from the people involved with the program how well they thought it was performing, what its strengths were, and what remained to be achieved. The lessons learned could be of use in other countries, particularly those recovering from civil conflict and other emergencies.

**What Did the Researchers Do and Find?**

In 2003, the researchers reviewed all available documents that had been written about the tuberculosis program. They also carried out interviews with 24 senior people involved with the program. Some of them were East Timorese, and some were from international organizations. The questions asked in the interviews were semi-structured. In other words, the researchers wanted to make sure that certain topics were covered but also wanted the people they questioned to have freedom in the way they gave their answers; they were not restricted to answering only “yes” or “no.” This kind of approach, where there is no gathering of precise figures that can be mathematically analyzed, is known as qualitative research.

The national tuberculosis program was considered to be working well in 2003. The researchers concluded that good coordination, cooperation, and collaboration were the most important factors contributing to the successes that had been achieved. The existing local structure and experience of the local NGO, the commitment among local personnel and international advisors to establishing an effective program, and the willingness of international advisers and local counterparts to be flexible in their approach were also important factors. The feeling among some government health workers that they lacked “ownership” of the program was one problem that still needed to be overcome.

**What Do These Findings Mean?**

Even after a major conflict, it was possible to launch an effective tuberculosis program in East Timor. Other countries in similar situations might be able to achieve success by applying the same approach. Unfortunately, renewed conflict broke out in East Timor in 2006. It will again be necessary to restore services, putting to use the lessons already learned.

**Additional Information.**

**Please access these Web sites via the online version of this summary at** http://dx.doi.org/10.1371/journal.pmed.0030383.

- Basic information about tuberculosis can be found on the Web site of the US National Institute of Allergy and Infectious Diseases
- The Web site of the World Health Organization's Stop TB department describes the recommended strategies for tuberculosis control
- TB Alert, a UK-based charity that promotes tuberculosis awareness worldwide, has information on tuberculosis in several European, African, and Asian languages
- A country profile of East Timor  is available on the BBC Web site

## Introduction

Tuberculosis (TB) infection and disease is intertwined with poverty [1]. The overwhelming burden of TB, over 80% of the 4.4 million cases of TB notified to the World Health Organization (WHO) in 2003, lies in developing countries [2]. Almost 60% of the cases were notified from Asian or Pacific countries [2].

In 1993, WHO declared TB a global emergency and formulated a “five-point plan” known as DOTS (for “directly observed treatment, short-course”) to fight the disease. It comprises political commitment, case detection using sputum microscopy, standardized short-course chemotherapy under proper case-management conditions including directly observed treatment, a regular drug supply, and a standardized recording and reporting system that allows assessment of individual patients as well as overall program performance [3]. Huge strides have been made in DOTS implementation during the past decade. There is a need for research to address the applicability of the DOTS strategy in post-conflict settings because of the often extraordinary circumstances present [1].

East Timor shares a border with Indonesia and lies 800 km to the northwest of Australia. It is one of the poorest countries in the Asia-Pacific. The population of 925,000 are predominantly subsistence farmers; their families live in rural areas [4]. After 400 y as a colony of Portugal, East Timor was annexed by Indonesia in 1975, and for the following 24 y it was considered a province of Indonesia. In 1999, the country was torn apart by political violence led by groups who opposed the outcome of a referendum in favour of East Timorese independence. Militia systematically ransacked, looted, and destroyed up to 70% of infrastructure in almost every town and village, with government buildings—including health facilities—being major targets. The health-care system collapsed. United Nations (UN) peacekeeping troops intervened and stabilised the country under UN administration for 2 y, before East Timor gained independence in May 2002 (Table 1).

**Table 1:**
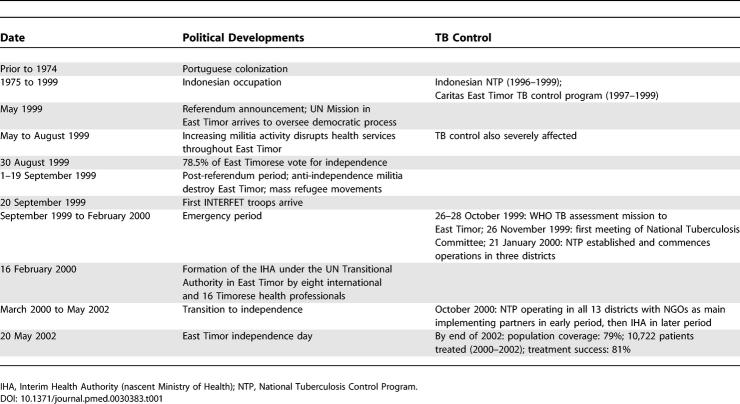
Timeline of Key Political and TB Control Developments in East Timor

The international humanitarian response to the complex emergency in 1999 was swift: within a week of the international sanction of a UN peace enforcement mission (International Force East Timor [INTERFET]) in September, several UN agencies (including WHO) had arrived. Within a month, around 100 international non-government organisations (NGOs), many with health components to their work, were present in the country. The lack of government structures, the absence of many community leaders, the mass displacement of the population, and a perceived reluctance on the part of East Timorese leaders to make decisions in these circumstances made dealing with the situation from a development perspective difficult [5]. In the emergency phase, health services were mainly delivered by international NGOs, with church-run clinics and some not-for-profit private practices providing important additional coverage. In the transition to independence, NGO-run health services were progressively handed over to the nascent Ministry for Health (the Interim Health Authority) under the UN administration (Table 1).

**Figure 1:**
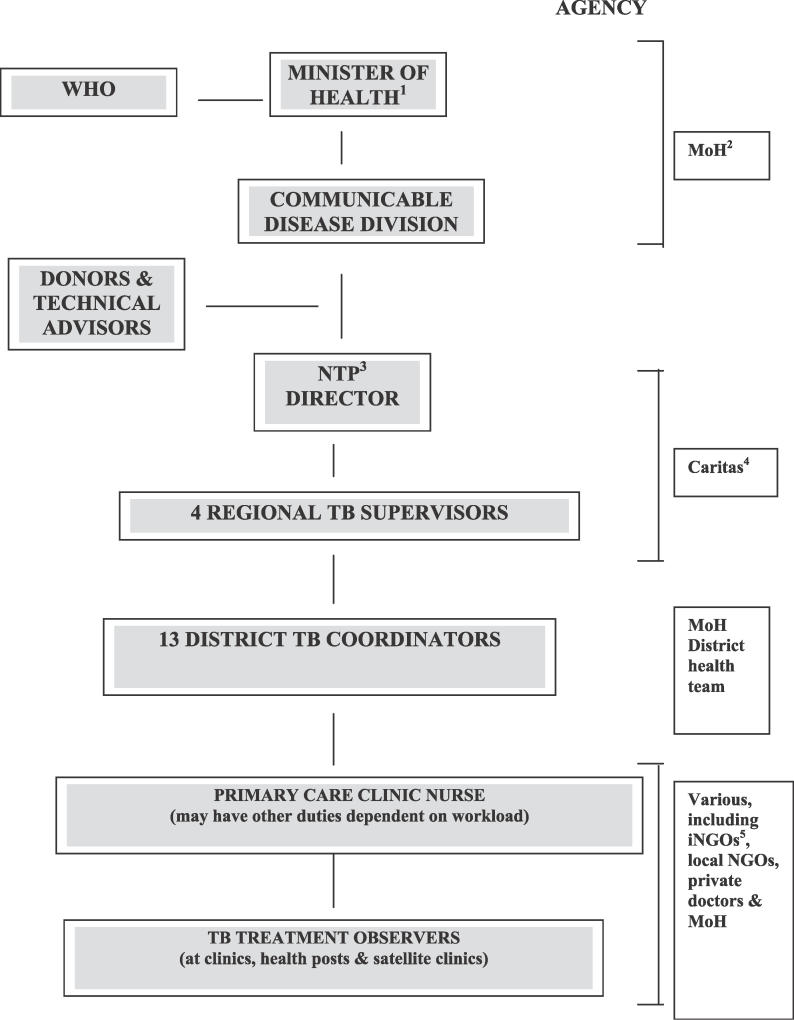
Structure of the National Tuberculosis Control Program in East Timor, 1999–2002 ^1^Minister of Health includes earlier health leaders, including the co-chair of the Interim Health Authority of the United Nations Transitional Administration in East Timor (UNTAET) and later Head of the Division of Health Services in the East Timorese Transitional Authority (ETTA). ^2^MoH, Ministry of Health. ^3^NTP, National Tuberculosis Control Program. ^4^Caritas, Caritas East Timor, a Timorese church-based NGO. ^5^iNGO, international NGO.

TB had, for many years, been identified as a major public health problem in East Timor. From 1996, two TB programs operated in parallel: one run by the Indonesian Ministry of Health and one run by an externally funded church-based organisation (Caritas East Timor). Following the referendum and associated violence, many health staff left East Timor or were unable to continue working, and TB services were severely disrupted [5]. The magnitude of the TB epidemic in East Timor is believed to have increased in 1999 [6]. Within a few months of the September events, people from many different organisations contributed to the establishment of a National Tuberculosis Control Program that conformed to WHO's DOTS strategy (Figure 1). In its first 5 y, the TB program diagnosed and commenced treatment for 17,210 patients [7]. In 2001, the notification rate was 446 cases per 100,000 population for all forms of TB, and 154 per 100,000 population for new smear-positive pulmonary cases. These are the highest rates in the Southeast Asian and Western Pacific Regions [2]. This reflects both the high burden of disease and the effective case-finding system that has been developed and sustained in East Timor since 1999. There has been gradual progress in DOTS expansion, with the TB program functioning in all 13 districts and accessible to 79% of the population of East Timor. Treatment results have improved, and the treatment success rate is now consistently over 80% [7].

This paper aims to identify the key factors that enabled the establishment of a successful TB program in East Timor, while attempts to develop other sustainable programs in the post-conflict period have struggled [5]. We document, analyse, and reflect on this set of experiences and place them in the public domain for critique and lesson-learning.

## Methods

The methods employed included the following: review of the published literature on post-conflict health systems development, review of documents and reports from the time period 1999–2003 relating to East Timor, interview of key informants with knowledge of the TB program and its development over this time period, and critical reflection on the experience of two key stakeholders and authors (N. M. and P. M. K.).

**Box 1. Insider and Outsider Perspectives in Qualitative Research**

An important feature of the work undertaken and presented here was the involvement of two of the authors in the events described in this paper. These insider–outsider perspectives are contentious, but we believe add considerable strength to the insights able to be derived through such work: they offer an opportunity to tell the story of the TB program through the words of both the authors and the range of informants consulted, and they demand reference back to the real constraints and opportunities operating in such environments. They add to the reflections of others to develop a rich insight into the perspectives of the stakeholders involved at the time, and their voices, heard through this paper.

The boundary between researcher and researched frequently blurs in qualitative studies [32]. The authors (N. M., the founding director of the TB program, and P. M. K., an international TB consultant) were key participants in the processes and events described in this paper. Power relationships are always problematic in research, in particular in qualitative research. We were acutely aware of this during the planning phase, the research itself, the analysis of the data, and the writing up of our findings. The principal author (N. M.) was the key to access in this study, and we feel that this unique access to such a wide variety of opinions outweighs the potential bias that his previous position may have bought to the study findings. It should be noted that N. M. was not in a position of power when the interviews took place (he had resigned as director of the TB program some 3 y previously). In Timorese society, it is true that social hierarchy can lead to those perceived as “inferiors” being reticent to criticise their “superiors”. However, peers and those in socially superior positions are typically frank with their opinions, as evidenced by the dissenting and critical statements included in the article. This free exchange of views is also demonstrated by the dissenting opinions obtained in interviews of foreign staff. Thus, we are confident that we have addressed this important issue, and acknowledged potential bias in our conclusions.

The positioning of two of the authors as “insiders”, particularly in combination with two “outsider” authors, adds strength, but also has some limitations. The limitations include whether too strong a voice is given to the particular perspectives of two of the players, and whether their involvement in conduct and analysis of interviews undermines objective assessment. The involvement of the two authors not involved in the TB program development offers an opportunity to engage with some of the material through review of the interview data, and to test out some of the ideas and the analysis.

As stated by Merriam and colleagues [33], “the insider's strengths become the outsider's weaknesses and vice-versa”, and “this multiplicity of perspectives” enhances rather than detracts from the validity of observations made. Developing an insider–outsider team helps establish balance. What is presented can perhaps be described as the perspectives and critical insights of key players in the establishment of the TB program at a crucial time in its history. The benefit is that unique insights are documented, analysed, and presented for further critique and analysis: no attempt is made here to state that the insights presented in this paper are neutral or not coloured by the involvement of two key players. Rather we argue that this history provides additional depth and richness, available for others to build upon.

All stakeholders and influential individuals currently and/or previously involved in the setting up and administration of the National Tuberculosis Control Program between 1999 and 2003 were identified and contacted with a request to interview them. Of the 30 people identified, 24 were available for interview. No stakeholder refused participation, and the six who did not participate were either non-contactable or were otherwise unavailable for interview. Stakeholders who were not interviewed were represented in the interviewed group by others who held closely related posts during the time period under study. Those who participated in this study included government officials (the Minister of Health and other high-ranking staff), local and international health professionals, staff of local and international NGOs involved in health services delivery, private practitioners, local politicians, church leaders, current and previous TB program directors and staff, the current and previous WHO representatives in East Timor, donor representatives, and TB consultants.

Semi-structured interviews were conducted by the principal author (NM), either face-to-face (*n =* 16) or via E-mail (*n =* 8). Open-ended questions were employed in order to gain as much qualitative information as possible about the participants' views on the process of establishing the National Tuberculosis Control Program. Participants were also asked to comment on the effectiveness of the TB program, and the current and future challenges it faces. The interviews were recorded in handwritten notes, tape recordings and E-mail messages in response to the interviewer's written questions. After each interview a summary transcript was prepared, and participants were offered the opportunity to read and check these for accuracy. Where required, follow-up interviews were undertaken to clarify particular comments and issues. From these interviews we were able to identify, through the perspectives of these stakeholders, the factors that were perceived to contribute to the success of the East Timor TB program, and the challenges it faces in the future. Three authors (NM, ABZ, and JAG) contributed to the stakeholder analysis, drawing on the methods described by Varvasovszky and Brugha [8]. Two of the authors (ABZ and JAG) were not directly involved with the events reported in this paper and acted as independent external observers within the team (see Box 1).

Ethical approval for the study was obtained from the Human Research Ethics Committees of the Menzies School of Health Research and of Charles Darwin University, Darwin, Australia. In the absence of a local ethics committee, written permission to carry out the study was obtained from the Minister for Health of the Democratic Republic of East Timor.

## Results

The key barriers to establishing the National Tuberculosis Control Program are summarised in Table 2. These included population mobility; lack of health infrastructure, supplies, equipment, and trained staff; transport and communication difficulties; and a scarcity of financial, technical, and political will to address TB in the emergency phase.

**Table 2:**
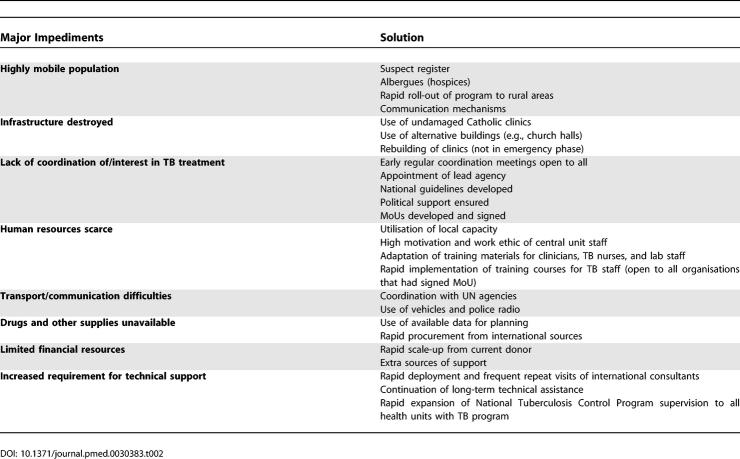
The Major Impediments to the Establishment of a National TB Control Program in East Timor, the Solutions Identified, and the Agencies Responsible for Their Implementation

Most participants expressed concern about the increased number of TB cases during the emergency period, most likely due to the massive displacement of people; malnutrition, sanitation, and shelter problems; and the disruption of the previous TB programs. WHO reported that there had been no TB treatment program for several months in late 1999, therefore there was a “backlog” of patients requiring treatment [9]. The displacement of the majority of the country's population was another major problem in the implementation of an effective TB treatment program. It was reported that there was a large population drift to Dili during the first few months of the emergency period, for security, food, shelter, and the possibility of gaining employment [9]. This situation greatly concerned some of the participants in this study, who during the first TB program coordination meeting predicted future constraints to the successful completion of TB treatment when these people returned to their villages after having begun treatment in Dili.

There were also a number of factors that delayed the process of establishing the TB program. Lack of money, drugs, infrastructure, and transportation characterised all sectors in East Timor in the early emergency period. It was very difficult to get from one place to another in order to coordinate the work without regular and reliable transport and amidst security concerns in some areas. There were many times when TB program staff had to rely on the UN and NGOs for transportation. In the early stage of the TB program, the program was mainly run through the Catholic Church clinics because many of these had not been totally destroyed, unlike government clinics.

The main enabling factors for success of the TB program are also summarised in Table 2. The findings of this study suggest that at the centre of the successful development of East Timor's TB program were coordination, cooperation, and collaboration between local and international agencies, TB experts, and staff. These were brought about by a willingness to take into account and adapt to local contextual factors (flexibility), and the strong commitment and high level of motivation on the part of key stakeholders.

### Coordination and Cooperation

By coordination, we mean a sharing of responsibilities to prevent a duplication of effort. Cooperation is a closely related concept, here referring to a sense of common purpose, and the use of common methods to work together to achieve something.

Most participants in this study considered coordination to be a major factor in the successful establishment of a national TB program post-conflict, while acknowledging that it was very difficult to achieve given the conditions of the early emergency phase. The competing interests of local and international health professionals, NGO, UN agencies, and military forces fought against it.

In the early phase of the emergency, more than 15 NGOs plus the International Committee of the Red Cross and the INTERFET military health team arrived to give assistance, each of them proposing to develop activities in a particular geographic region or in relation to a particular component of health policy and system development [10]. WHO attempted to act as a coordinating body for health but experienced a number of constraints. Some of those interviewed expressed the opinion that budget limitations had prevented WHO from functioning as effectively as had been hoped for. At that time, services including health were highly centralised, and health service teams were sent out from Dili to different parts of the country in the company of the security forces. Neither capacity building nor institutional development occurred [5,10].

Soon after the conflict in 1999, TB was regarded as a relatively minor and non-urgent issue compared to diseases with high outbreak potential such as cholera, dysentery, dengue fever, and measles. Most of the international NGOs chose not to include TB in their treatment procedures. As one participant explained:

The international NGOs had their own mission, and they focused more on acute or emergency cases. Although they were aware of the increasing number of TB cases, they did nothing because there was no mechanism through which they could do so. [Senior TB program official]

The Caritas TB program was reported to be still functioning in some places at this time, and there were also some uncoordinated, and some might say inappropriate, TB treatments being provided by local health professionals, who administered single-drug treatment to TB patients. These local professionals had obtained the drugs from government health centres before they were destroyed by militia during the period of violence. It was difficult for the National Tuberculosis Control Program to convince these local professionals to follow international best practice in treating TB patients because they had been following a different treatment regime for a long time. Some international medical practitioners also followed sub-optimal treatment regimens.

During the emergency period, there was a vacuum. There was no coordination. Everybody felt they had the right to treat TB patients without follow-up because they had no capacity to follow up the patient. There wasn't good coordination. [Founding member of the Interim Health Authority]

Of those NGOs treating people for TB, not all followed the WHO recommended treatment schedule [9]. International NGOs and certain “independent” international staff maintained strong views on how TB patients should be treated. On the one hand, they insisted on their own procedures for TB control, and their “right” to diagnose and treat TB patients without follow-up in many cases. On the other hand, some refused to diagnose and treat TB, stating that it was not a priority in a complex emergency situation, and their organisations' mandates precluded them from doing so.

Barriers to implement [the National Tuberculosis Control Program] were obviously plenty, but what struck me most was the difficulty in coordinating health professionals, particularly to make them adhere to the [East Timor National Tuberculosis Control Program] Manual or standard protocol. This is particularly so in the case of medical doctors who came from a wide gamut of countries in the world, bringing their own preferences and practices while ignoring the international standards of DOTS adopted by the TB program. [Senior Ministry of Health Official]

In establishing a policy framework for the provision of general health services to the population, many interviewees reported that, at least in the emergency period (see Table 1), there were major difficulties in coordination between WHO, the United Nations Children's Fund, NGOs, local professionals, the East Timorese Interim Health Authority, and bilateral and multilateral donors at the highest level. Key barriers to coordination were competition (between organisations and individuals) and differences of opinion on priorities. This included competition for resources (financing and local personnel), legitimacy, and leadership. For example, some donors and some NGOs wanted to introduce their own style of TB control without reference to the local situation.

Despite this there was intensive negotiation in an effort to coordinate the work for the establishment of an effective TB program. There was strong commitment to preventing the re-establishment of parallel TB programs. Experience from the Indonesian period demonstrated that a coordinated approach was crucial.

The experiences of TB treatment during the Indonesian period inspired us to strengthen the coordination and control of the treatment strategy. We [the Interim Health Authority] appointed Caritas as the sole implementer of the TB program in East Timor, with the authority to ban private practitioners or NGOs from treating TB cases and importing TB drugs without their permission. [Founding member of the Interim Health Authority]

Caritas East Timor began holding weekly coordination meetings to accelerate the establishment of the TB program in late 1999. Local and international professionals, local and international NGOs working in health, clinicians, donor representatives, other UN agencies such as the World Food Program and the United Nations Children's Fund, and large NGOs such as the International Organization for Migration, the International Committee of the Red Cross, and Oxfam were among those who routinely attended. These meetings led to a high degree of cooperation from the organisations involved. Senior staff made a commitment to come to these meetings that was vitally important for the planning process. Continuity and consistency of decision-making and the ability to carry this back to the field was also important. A similar phenomenon was observed outside of Dili as the TB program rolled out to the rural districts. The agreements on roles and responsibilities formulated in these early coordination meetings eventually resulted in a series of memoranda of understanding (MoUs) between the TB program and the implementing partners that codified the responsibilities of the agencies involved in the implementation of the TB program in the emergency and early transition period. These MoUs were crucial in enforcing a standardised national approach to TB control, particularly in the absence of any legislative framework.

Cooperation between international TB consultants and their Timorese colleagues as characterised by the willingness to adapt to local circumstances was an important factor in the establishment of a sustainable TB program in East Timor. The international consultants involved did not see East Timor as a “blank slate” upon which their public health plans could be written without reference to local conditions. The previous TB control structures and the experience and expertise of local partners were acknowledged and became central to the establishment of the TB program. From the outset, the opinions of local TB control staff were sought before any decisions were made on technical and operational aspects. At their second meeting, the coordinating committee of the TB program appointed a Timorese doctor (NM) as the national TB coordinator, who chaired all subsequent meetings and became the founding TB program director.

We had the money and expertise; we could easily have built a sophisticated, though unsustainable TB program in East Timor. However, we did not do it because we did not want to be involved in the same mistakes as has occurred in Kosovo and Cambodia. [international TB consultant]

In East Timor, money and drugs became readily available because of the prompt response to the crisis by donors, including the long-term donor to the NGO program. This continuity of financial and technical support, together with additional support from other sources in the emergency period, was identified as one of the most important factors in accelerating the process of developing the TB program.

### Collaboration

Collaboration is a concept that is deeper than cooperation and represents a shared vision for the future and not merely the absence of competition. Partnership, shared understanding, open communication, tolerance of difference, and trust are key components of a collaborative approach. In the case of the National Tuberculosis Control Program in East Timor, successful implementation depended on the readiness of local staff to trust their international counterparts, to learn from them, and to adopt the international best practice standard. At the time, many East Timorese were experiencing intense feelings of distrust following the deeply disturbing and destructive events of September 1999. The withdrawal of the United Nations Mission in East Timor (UNAMET) at the time of the violence despite earlier commitments to protect voters after the referendum, regardless of the result, reflected badly on international staff. Many members of the UN mission were surprised to experience rejection from their East Timorese colleagues. The lack of trust needed to be managed sensitively and sincerely. Sergio Lobo—the Interim Health Authority Co-Chair at the time—coined the phrase “one table, two chairs” to encapsulate the expectation that local and international counterparts would sit and work together, to develop ideas and make decisions. This was central throughout the process of developing the TB program.

Well, I think it is because there was a willingness to work together between local and international expertise. But it depends on the international expert or counterpart to transfer the knowledge and provide a motivation [to the local staff]. On the other hand, the willingness of local professionals to accept the opportunity to learn and to work based on the international best practice standard [is also important]. [senior TB program official]

Throughout, there was also strong commitment from TB program consultants and staff to incorporate international best practice guidelines at the local level. This was covered in a MoU signed by the main stakeholders in early 2000 (Table 3).

**Table 3:**
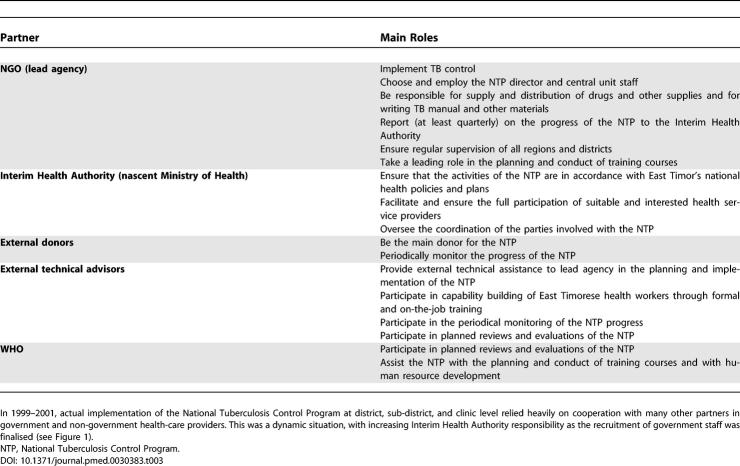
Main Roles of Collaborating Partners in the Establishment and Implementation of the TB Program in East Timor, as Laid out in MoU in 2000

One strength of the collaboration at that time was the ability of international experts to recognise the importance of the National Council of East Timorese Resistance (CNRT) as the only representative political organisation in East Timor.

At my first attendance as CNRT representative to the health coordination meeting organised by WHO and UNICEF [United Nations Children's Fund], I realized that the meeting was to coordinate the work of the international NGOs and agencies. Later on, I was told that the UN and most of [the] international agencies have a mandate or regulation that prevents them to work with any political party. CNRT has been regarded as a political party. [CNRT health spokesman]

There were strong links between the CNRT and Caritas East Timor during the Indonesian period. This internal political profile, Caritas East Timor's track record in TB control, and the recommendations of the October 1999 WHO assessment mission prompted the Interim Health Authority to appoint Caritas East Timor as the lead agency in the new National Tuberculosis Control Program [11]. This decision was crucial to establishing a sustainable TB program.

The government [Interim Health Authority] made a decision to have only one TB program and appointed Caritas East Timor as the implementing agency. That decision was based on: firstly, because [of] the fact that the Catholic clinics structure was there; secondly, Caritas East Timor has experienced staff who were willing to run and manage the program; and lastly, the government didn't yet have the structure to manage and run the program. [founding member of the Interim Health Authority]

East Timorese sovereignty over the TB program was maintained throughout the establishment and expansion of the TB program. This ensured a rapid response by the TB program leadership, with the assistance of international consultants, to feedback from the field, and to the analysis of quantitative data available from the program.

Most of the participants in this study felt that staff motivation was also a key factor in the success of the TB program. This was a difficult time for the staff in the early stage, with most directly or indirectly affected by the post-referendum violence. Despite this, most TB control staff remained highly motivated and committed. The Christian ethos of the NGO staff was an important factor, and staff drew strength from their Christian beliefs to serve those in need. The strong Timorese leadership was also an important motivating factor. There was the previous experience from the NGO TB program pre-independence, there was also a certain amount of euphoria about finally becoming independent, which led to many extraordinary things. Nation building, contributing from one's own expertise, coming together to assist fellow Timorese, these were all elements that bolstered health workers working for no pay, or only minimal pay, during the emergency and even well into the post-emergency phase.

In January 2000, less than 2 mo after the first coordination meeting, National Tuberculosis Control Program was launched. This occurred 1 mo prior to the establishment of the Interim Health Authority. Five months after the first meeting, one co-chair of the Interim Health Authority referred to the TB program as the “shining light” in the reconstruction process of East Timor, a view supported by many interviewees.

It [the TB program] was the first program to develop a memorandum of understanding....In that sense it was a model of collaboration in which all of the partners had clearly defined roles. The links with the church-run health services [were] a great asset in re-establishing the program as well as the continuous commitment of Caritas Norway. The proximity of the technical assistance in Darwin was obviously helpful. [founding member of the Interim Health Authority]

### Four Years On: Current Challenges for the TB Program in East Timor

TB control remains a public health priority for the new East Timorese administration. Participants in this study are aware of many problems that might affect the program's future effectiveness and sustainability.

Indeed, the TB program [is] probably facing the same constraints that TB programs everywhere in less developed countries are facing: personnel issues, supervision, quality control, etc. [founding member of the Interim Health Authority]

The lack of a sense of ownership of the program by government health staff has been noted since the TB program launch. Some staff of government clinics still regard the TB program as a non-government program and refuse to commit their time. The perception that the TB program is the responsibility of Caritas has begun to change since the Minister of Health wrote a letter to staff directing them to cooperate with the TB program as part of the core business of the Government Health Service. However, this cooperation needs further consolidation through the involvement of sub-districts and health posts in all TB program activities [7].

There has been a feeling of job insecurity and dissatisfaction among TB program staff due to the fact that many are on short-term contracts with NGOs. In some cases, those with previous experience in TB have been appointed to non-TB-program positions in the government health service, leaving their positions in the TB program vacant. Consequently, the government has had to recruit staff that have no previous TB program experience for key roles within the TB program. This has necessitated retraining in many cases.

The government has been recruiting the experienced staff to work in the TB program; however, because of their competency they are often promoted to higher level positions, leaving their [empty] posts to be filled by those who lack TB experience. [senior Ministry of Health official]

Some participants felt there is a need to change the government recruitment procedure so as to keep the current TB program structure under Caritas. Given continuing capacity gaps in the government health service, they felt that Caritas is the best organisation to continue to manage the TB program. However, there is a recognised need for the government to take over some coordination and financial responsibility so as to gain a greater sense of ownership of the program. Navigating this challenging period of handover from NGO to government sensitively and constructively is key to the long-term sustainability of most, if not all, emergency-introduced health programs.

There was also concern expressed about the low level of community awareness of TB in East Timor, and some argued that unless this issue is addressed TB will always be a problem. Some informants suggested that this problem could be overcome by conducting a promotional campaign on TB to increase patient and community awareness. It is a challenge not only to explain what the TB program strategy involves but also to help patients understand the rationale behind the strategy. As one participant argued:

There is a need to allocate more time and resources to conduct [a] nation-wide health education campaign in the country. This will reduce stigma and improve the patients' knowledge on TB. [international TB consultant]

Major challenges remain in determining the appropriate balance between different components of TB programs and in recognising the limitations of each component in achieving long-term sustainable health outcomes.

## Discussion

There have been consistent calls for a more reflective approach to the evaluation of health-care interventions in conflict-affected settings—leading to policies and guidelines better informed by evidence—if we are to improve the international response [12–15]. Several recent high-profile complex emergencies—in Mozambique, Cambodia, Kosovo, East Timor, Afghanistan, and Iraq—demonstrate that post-conflict situations, and the response to them, as well as the response required, have changed in a number of important ways. TB control in these settings is particularly interesting and illustrative.

The geographic distribution of TB and that of political conflict overlap. Of the top 22 countries with high TB burden, which account for 80% of all cases of TB in the world, seven have a history of significant conflict and/or complex emergency situations in recent years [2]. During the 1980s and 1990s, high rates of TB disease and TB mortality were reported in refugee camps in Africa and Asia [16–20]. Social and political disruption fuel TB epidemics and disrupt the delivery of TB control services, resulting in increased numbers of cases and, because of incomplete treatment, heighten the possibility of multiple-drug-resistant TB [21,22].

This global epidemiology reinforces the need to improve TB control in these complex settings, both for the benefit of the people affected by the conflict and for neighbouring nations, including those that receive refugees for temporary or permanent re-settlement. This was dramatically demonstrated by the high rate of TB documented in participants of the “safe haven” exercise in East Timor in 1999 [6]. East Timorese arriving in Darwin during September 1999 represented less than 0.01% of the Australian population, but accounted for 6% of all TB cases diagnosed in Australia in that year [23].

Continuity of health systems in periods of transition from pre-emergency to during emergency to post-emergency and ultimately to sustainable programs is an important component of international responses to emergencies, but is rarely achieved. Coordination, cooperation, and collaboration were key components to achieving this aim in relation to TB control in East Timor.

### Coordination

Poor coordination is a well established problem in the response to complex emergencies [13,21,24]. As was the case in Kosovo, WHO's ambition to become the implementing agency for certain programs meant that it was at risk of being seen as a competitor with other implementing agencies [13]. Constrained organisational mandates have been described elsewhere as a potential impediment to comprehensive care and services [25]. It can be argued that this is a reasonable and perhaps responsible attitude for agencies with short-term commitments and a lack of expertise in TB. However, in the context of East Timor in 1999, many of the usual public health and clinical concerns of emergency situations were not present, whilst TB was so prominent [6,26]. It was thus appropriate that the health sector did treat TB as a priority issue in the early emergency phase.

Caritas East Timor was appointed as the lead agency of the National Tuberculosis Control Program and successfully acted as the coordinating body. The role of faith-based NGOs is increasingly recognised as important—but not without complexity and contradictions—in international development settings [27]. One of the negative aspects of having leadership be in the hands of an NGO is the NGO's lack of legislative authority. Hence, cooperation with the government is crucial. For example, in East Timor, the TB program required the Interim Health Authority to take a policy decision to stop the private sector from importing TB drugs or treating TB independent of the TB control program.

### Cooperation

The emergency period is critical. The rapid injection of money, expertise, and technology makes the recipient countries vulnerable to the development of sophisticated programs that they will not be able to sustain. It takes good planning with extensive knowledge of the local situation to establish a cost-effective and sustainable program for a country under these circumstances, and cooperation, particularly with local counterparts, is a key component for facilitating this desirable outcome. The inclusive approach taken during the establishment of the TB program in East Timor was in contrast to some other programs introduced in the emergency period by specific donors and/or international NGOs. The Roll Back Malaria campaign [28] and an oral health program [5] lacked the same level of cooperation with local partners, and these programs have been less successfully sustained in the longer term. In Cambodia the UN took control of the TB Control Program in 1989 and sidelined local expertise for political reasons, allowing state-based structures and services to be unwound [29]. The result was limited short-term success, but an unsustainable program in the long term [29].

### Collaboration

The truly collaborative approach used in the establishment of the TB control program in East Timor was built on mutual trust and flexibility. Key to the development of these was a recognition of the political context of East Timor by international TB advisors.

In contrast with other political emergencies, for example, in Kosovo and Cambodia, where competing political interests were prominent and contestation the norm, [13] the CNRT was the single, uncontested political authority in post-conflict East Timor. While the CNRT was the umbrella organisation for all political parties and groups that fought for independence, in the early phase of the emergency period most of the NGOs and UN agencies refused to work with them as they were bound by their mandate not to deal with political parties. This created a sense of exclusion among the Timorese. WHO was the exception, and considered the CNRT a valuable partner in health system development [26].

International technical advisors truly listened to local experience and accepted that this experience was valid in the local context of East Timor. Local solutions, not just the uncritical adoption of international standard solutions, were key components of the success of the TB program [30]. This flexible approach was more about the maintenance of Timorese sovereignty and the recognition of the local political context than about radical changes to internationally recognised best practice guidelines. All of the essential elements of DOTS were maintained, with the adoption of some local adaptations (e.g., use of hospices in the intensive phase of treatment, a network of satellite clinics in churches and other public buildings, and the use of incentives to ensure treatment compliance) [7]. Trust is increasingly being described as a key issue in the re-establishment of services in the aftermath of conflict [31]. For Timorese health workers, the mixture of feelings of euphoria, pride, and distrust might have prevented them from accepting and cooperating with the international staff in the initial post-conflict period. Importantly, local TB health workers trusted their international advisors and heeded their advice concerning, for example, the need for an inclusive approach and the use of MoUs. Thus, flexibility, built on trust, was a crucial two-way process in the TB control program establishment.

### Conclusion

During 2000, a sustainable national TB control program was established in East Timor in a post-conflict setting, despite the many difficulties encountered. Coordination, cooperation, and collaboration were the keys to this success, achieved through the high level of commitment of those involved, and the willingness of international experts and agencies to acknowledge local expertise and adapt to local circumstances. The latter was most evident in the early appointment of a local NGO to manage and implement the program. The utilisation of an existing local structure drawing on local expertise and experienced staff was essential to the program's success. The East Timorese TB program continues to function well and to improve the breadth and quality of its interventions. The government's commitment to, and sense of ownership of, the national TB program will be important to its continued performance improvement, and to ensuring its long-term sustainability. It is acknowledged that certain contextual issues, including the relatively small size of the country, the presence of charismatic Timorese leadership, and the presence of ongoing external financial and technical support, have also contributed to the establishment of the TB program. The East Timorese experience holds useful lessons for international donors and aid agencies and for those establishing TB control programs in other complex emergency situations in the future.

## Supporting Information

Alternative Language Abstract S1 Indonesian Translation of the Abstract (23 KB DOC)

Alternative Language Abstract S2 Tetun Translation of the Abstract (23 KB DOC)

## Abbreviations

CNRT: National Council of East Timorese Resistance
INTERFET: International Force East Timor
MoU: memorandum of understanding
NGO: non-government orgnanisation
TB: tuberculosis
UN: United Nations
WHO: World Health Organization
