# Supplementary material for: Reconstructing Tuberculosis Services after Major Conflict: Experiences and Lessons Learned in East Timor
Source: PLoS Med. 2006 Aug 22;3(10):e383. doi: 10.1371/journal.pmed.0030383 (PMC1550733; doi:10.1371/journal.pmed.0030383)
Supplement: Supplementary file 1 [file pmed.0030383.sd001.doc]

# SUMMARY (INDONESIAN)

Tuberkulosis (TBC) adalah masalah kesehatan masyarakat utama di negara-negara berkembang. Setelah hancurnya sistem kesehatan di Timor Leste yang disebabkan keganasan konflik politik pada tahun 1999, program nasional pengontrolan tuberkulosis dibentuk pada sebuah lembaga swadaya masyarakat (LSM) yang berfungsi sebagai agen penuntun. Dalam waktu beberap bulan, program pengontrolan TBC telah berfungsi diseluruh kabupaten. Menggunakan pengalaman Timor Leste sebagai studi kasus, kami telah menguji faktor-faktor pendukung dalam pengimplementasian program pengontrolan penyakit menular ini dalam situasi paskah konflik. Kami melakukan analisa terhadap pihak-pihak terkait (stakeholder) dan mengadakan semi interview yang terstruktur dengan 24 pihak terkait, lokal maupun internasional pada tahun 2003. Koordinasi, koperasi (kerja-sama) dan kolaborasi diidentifikasi sebagai kontributor utama pada kesukseksan program TBC. Tersedianya struktur lokal, pengalaman dari LSM lokal, komitmen diantara tenaga lokal dan penasehat internasional untuk membentuk suatu program yang efektif, dan kemauan dari penasehat internasional dan tenaga lokal untuk tidak bersifat kaku pada pendekatannya juga merupakan faktor yang penting. Kesuksesan ini dicapai meskipun menghadapi beberapa hambatan utama, termasuk perpindahan penduduk yang besar, kekurangan infrastruktur, dan persaingan kepentingan dari organisasi-organisasi yang bekerja pada sektor kesehatan. Lima tahun setelah konflik, program pengontrolan TBC terus beroperasi di seluruh kabupaten dengan angka pelaporan kasus yang tinggi, walau perasaan kepemilikan terhadap program TBC dari pegawai pemerintah masih merupakan tantangan. Pengetahuan yang didapat di Timor Leste kemungkinan dapat di pakai di situasi-situasi paskah konflik lain yang memiliki angka prevalensi TBC yang tinggi dan mungkin juga relevan untuk program-program pengontrolan penyakit yang lain.
