# Supplementary material for: Reconstructing Tuberculosis Services after Major Conflict: Experiences and Lessons Learned in East Timor
Source: PLoS Med. 2006 Aug 22;3(10):e383. doi: 10.1371/journal.pmed.0030383 (PMC1550733; doi:10.1371/journal.pmed.0030383)
Supplement: Supplementary file 2 [file pmed.0030383.sd002.doc]

# SUMMARY (TETUN)

Tuberculosa (TB) ne’e problema saude publika boot iha nasaun ladun desemvolve sira. Hafoin de destrusaun de sistema saude Timor Leste relasiona ho violensia konflitu politika nebe akontese in 1999, programa nasional kontrola tuberkulosa estabilisa iha organisasaun non govermentais (ONG) nia okus, nebe sai mos hanesan ajensia lenodalan. Iha fulan hira nia laran deit, programa TB fungsiona no operasional iha distritu hotu. Utiliza programa TB Timor Leste hanesan casu de estudo, ami examina fatores-fatores nebe asisti iha implementasaun moras kontaqiosa ida ne iha situsaun post-konflitu. Ami halao analisa ho parseiros involvidu (stakeholder) no konduta semi-struturadu intervista ho 24 parseiros involvidu, lokal no internasional iha tinan 2003. Ami identifika koordenasaun, koperasaun no kolaborasun hanesan fatores maioria konrtibui ba suseso program TB ida ne’e. Existensia strutura lokal no esperiensia husi ONG lokal, komitmento husi staf lokal no advisor internasional atu estabilisa program efektivu ida no intensaun diak husi advisor internasional no parseiro lokal atu iha flesibilidade iha sira nia intervensaun sai mos hanesan fatores importante ida. Suseso ne’e hetan maski hasoru desavio barak, inkluido disloksaun maioria husi populasaun, laiha infrastrutura, no kompetisaun interese husi organisasaun-organisasun nebe servisu iha area saude. Tinan lima hafoin de konflitu, program TB kontinua funsiona iha distritu hotu no fo dadus notifikasaun TB nebe as. Maski nune, funsionario publiku barak mak seidauk sente program TB hanesan programa governu nian, no ida ne’e sei sai hanesan disavio boot ida. Leisaun hetan iha Timor Leste karik bele aplika iha situasaun post konflitu sira seluk nebe iha dadus prevalensia TB as, no karik, bele mos iha relevansia ho program kontrola moras seluk.
